# Immediate neurophysiological effects of transcranial electrical stimulation

**DOI:** 10.1038/s41467-018-07233-7

**Published:** 2018-11-30

**Authors:** Anli Liu, Mihály Vöröslakos, Greg Kronberg, Simon Henin, Matthew R. Krause, Yu Huang, Alexander Opitz, Ashesh Mehta, Christopher C. Pack, Bart Krekelberg, Antal Berényi, Lucas C. Parra, Lucia Melloni, Orrin Devinsky, György Buzsáki

**Affiliations:** 1 New York University Comprehensive Epilepsy Center, 223 34th Street, New York, NY 10016 USA; 2 Department of Neurology, NYU Langone Health, 222 East 41st Street, 14th Floor, New York, NY 10016 USA; 3 MTA-SZTE ‘Momentum’ Oscillatory Neuronal Networks Research Group, Department of Physiology, Faculty of Medicine, University of Szeged, 10 Dom sq., Szeged, H-6720 Hungary; 4 New York University Neuroscience Institute, 435 East 30th Street, New York, NY 10016 USA; 5 Department of Biomedical Engineering, City College of New York, 160 Convent Ave, New York, NY 10031 USA; 6 Montreal Neurological Institute, McGill University, Montreal, QC H3A 2B4 Canada; 7 Department of Biomedical Engineering of Minnesota, 312 Church St. SE, Minneapolis, MN 55455 USA; 8 Department of Neurosurgery, Hofstra Northwell School of Medicine, 611 Northern Blvd, Great Neck, NY 11021 USA; 9 Feinstein Institute for Medical Research, Hofstra Northwell School of Medicine, 350 Community Drive, Manhasset, NY 11030 USA; 10 Center for Molecular and Behavioral Neuroscience, Rutgers University, 197 University Avenue, Newark, NJ 07102 USA; 11 Max Planck Institute for Empirical Aesthetics, Grüneburgweg 14, 60322 Frankfurt am Main, Germany

## Abstract

Noninvasive brain stimulation techniques are used in experimental and clinical fields for their potential effects on brain network dynamics and behavior. Transcranial electrical stimulation (TES), including transcranial direct current stimulation (tDCS) and transcranial alternating current stimulation (tACS), has gained popularity because of its convenience and potential as a chronic therapy. However, a mechanistic understanding of TES has lagged behind its widespread adoption. Here, we review data and modelling on the immediate neurophysiological effects of TES in vitro as well as in vivo in both humans and other animals. While it remains unclear how typical TES protocols affect neural activity, we propose that validated models of current flow should inform study design and artifacts should be carefully excluded during signal recording and analysis. Potential indirect effects of TES (e.g., peripheral stimulation) should be investigated in more detail and further explored in experimental designs. We also consider how novel technologies may stimulate the next generation of TES experiments and devices, thus enhancing validity, specificity, and reproducibility.

## Abstract (web summary)

Transcranial electrical stimulation techniques, such as tDCS and tACS, are popular tools for neuroscience and clinical therapy, but how low-intensity current might modulate brain activity remains unclear. In this review, the authors review the evidence on mechanisms of transcranial electrical stimulation.

## Introduction

Electrical stimulation to the brain has a long history in both science and medicine. In 1867, Helmholtz discussed how electrical currents applied to the head can generate visual sensations^1^. Currents as low as 0.3 mA could induce phosphenes, and stronger currents could induce brighter and more lasting visual effects^2^. Subsequent studies confirmed that these visual phenomena result from retinal rather than direct brain stimulation^3,4^.

Early noninvasive electrical stimulation technologies used high intensities to directly affect brain activity (see Box 1). Electroconvulsive therapy, introduced into psychiatry in the 1930s, used currents of up to 60 mA to induce generalized seizures^5^. Subsequent studies on electroanesthesia and electrosleep used subconvulsive current intensities, delivered through large copper or platinum disks covered with saline-soaked gauze over frontal and occipital scalp, to affect large neocortical regions. To induce anesthesia, up to 40 mA direct currents (DC) or alternating currents (AC; 1 Hz–200 Hz) were used (See Box 1), while weaker intensities (3–10 mA) were typically needed for sleep induction^6^. More than 500 human surgeries were carried out under electrical anesthesia supplemented with medication^7^, but serious side effects led to the decline of the technique.

The remaining present-day application of high-intensity transcranial electrical stimulation (TES, see Box 1) is for intra-operative neuromonitoring. This technique, introduced by Merton and Merton (1980), uses high-intensity stimulation (up to 2000 V) through a pair of electrodes positioned over primary motor cortex to generate a visible twitch in the contralateral hand to monitor the functional integrity of central motor pathways during resective surgery^8–10^. These early applications for high-intensity TES^6,11,12^ laid the groundwork for subsequent experiments which suggested that weak currents applied to the scalp can also induce behavioral effects but without side effects and without conscious awareness of the stimulation^13,14^. Compared to other noninvasive neuromodulatory techniques such as transcranial magnetic stimulation (TMS) and ultrasound stimulation^15^, advantages of TES include low cost, portability, and potential in-home applications, fueling a proliferation of human trials^16,17^. However, a disadvantage of TES is that it may activate excitable peripheral elements between the scalp electrodes, including trigeminal nerve branches, the greater occipital nerve, retina, and vestibular organs.

Despite more than 4000 publications (PubMed) on TES in the past decade, we lack a mechanistic understanding of the mechanism (or mechanisms) by which this technique produces beneficial or deleterious effects. Most TES studies place an electrode above a targeted cortical region with the assumption that the underlying neuronal activity will be boosted or suppressed. While experiments in intact animals and computational models have studied physiological and behavioral effects of TES, the mechanisms are incompletely understood and their translation to humans even more uncertain. It is also unclear whether TES works through direct or indirect effects (see Box 1). This mechanistic uncertainty explains why behavioral effects in humans are often weak, variable and difficult to replicate^18,19^.

In this Review, we discuss the immediate (i.e., acute, concurrent, or online, see Box 1) neurophysiological effects of electrical field stimulation in brain slices, rodents, non-human primates, and humans. We compare experimental findings with computational models, highlight the gaps in understanding, and discuss novel methods to deliver spatially precise stimulation at higher intensities than conventionally used, while reducing unwanted peripheral effects. We close with recommendations for future clinical and translational TES investigations. We do not discuss here potential offline effects of TES, i.e. those effects which occur or persist after the current has been switched off (see Box 1). These have been reviewed elsewhere^20,21^.

## Biophysics of induced electric fields

At each point within the brain, a scalar electric potential can be measured relative to an arbitrary reference and expressed in Volts (V). The electric field $\vec E$ is the local change (gradient) of the voltage; $\vec E$ is a vector whose amplitude is measured in Volts per meter (V/m). All transmembrane currents from nearby neuronal processes contribute to generate $\overrightarrow E$ ^22^.

When a current is applied to brain tissue (as in TES), it affects the polarization of cellular membranes, which in turn can alter neuronal excitability. Terzuolo and Bullock^23^ first demonstrated this using DC stimulation applied with metal bars to the crayfish abdominal receptor and lobster cardiac ganglion neurons. Induced electric fields as low as 1 V/m affected the timing of action potentials while much higher fields triggered action potentials in silent neurons^23^. However, the orientation of the field was also important: rotating the applied electric field’s orientation relative to the main neuron’s soma-dendritic axis (e.g., from parallel to perpendicular to the main neuronal axis) affected the strength and direction of the effects. These findings generalize to the mammalian brain. An anode on the pial surface (cathode at depth) depolarizes neurons and increases neuronal firing frequency, while the reversed current flow (surface cathode, depth anode) hyperpolarizes neurons and reduces their firing rates^24,25^.

**Fig. 1:**
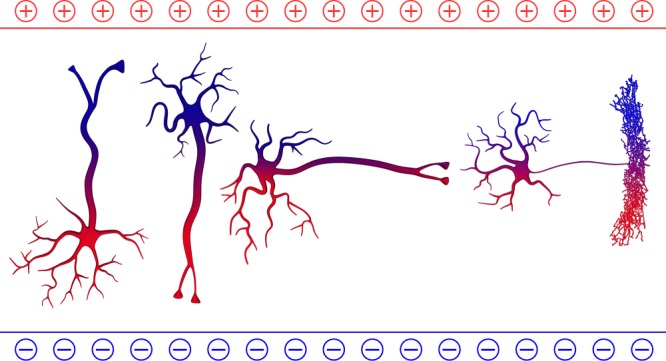
The impact of orientation of neuronal compartments on TES-induced excitability. Four idealized neurons are shown with different orientation relative to the induced electric field. The electric field can affect the soma, dendrites, axon initial segment and the axon tree differently. The relationship between the electric field vector and the morphology/orientation of neurons and individual neuronal compartments determine whether the neuron will be net depolarized or hyperpolarized

Yet, the above picture is an oversimplification. First, it assumes that the soma is singularly affected, but axon initial segment, dendrites, and axon are also affected with different, unknown sensitivities to electric fields. These combined and variable effects on neuronal compartments can alter neuronal spiking^19^. In addition, tonic depolarization of axon terminals could release neurotransmitters in an axon branch-specific manner without generating action potentials. Second, although fields perpendicular to the soma-dendritic axis may have little influence on the apical dendrites of pyramidal neurons^26–28^, they can activate basal dendrites and dense local axonal arbors of basket and chandelier interneurons. In addition, neurons with star-shaped dendrites—many inhibitory interneurons, layer 4 stellate cells, thalamocortical cells, and basal ganglia neurons—might be affected equally by all field orientations. These complexities (Fig. 1) are relevant because the orientation of TES-induced intracranial fields relative to these different neuronal populations and compartments varies considerably^29^. In summary, the influence of TES current depends not only on the amplitude but upon how the induced electric field relates to the three-dimensional organization of neuronal compartments^28,30,31^. Biophysical experiments and detailed computational models are needed to fully understand these complexities.

### Box 1 Terminology

Transcranial electrical stimulation (TES)—The technique of applying an electric field at the scalp surface with the purpose of directly affecting brain activity. Early efforts applied fields at high intensities for electroconvulsive therapy (ECT, 60 mA), electroanesthesia (40 mA), and electrosleep (3–10 mA). More recent efforts have applied TES at lower intensities (typically 1-2 mA) to reduce peripheral side effects, such as skin sensation and phosphenes.

Direct current stimulation—Electrical current applied in a constant, unidirectional manner flowing from anode to cathode. When DC stimulation is applied across the scalp, it is commonly referred to as transcranial direct current stimulation (TDCS).

Alternating current stimulation—Electrical current applied in a varying, typically sinusoidal, waveform, with current flowing from anode to cathode in one half-cycle and in the reverse direction in the second half-cycle. When the AC stimulation is applied across the scalp, it is commonly referred to as transcranial alternating current stimulation (TACS).

Electric field—The difference in voltage between two locations (in the brain). The electric field is a vector with both magnitude and direction, and is measured in units of Volts/meter (V/m).

Direct effects—Potential effects of TES on the excitability of neurons in the brain, which include both immediate and cumulative effects.

Indirect effects—Potential side effects of TES on non-neuronal elements, including placebo, peripheral nerves, retina, cochlea, glia, immune system, and blood flow as well as cumulative, long-term metabolic effects on neurons.

Immediate effects—Potential effects of TES which occur simultaneously or acutely during stimulation. These effects are measured by changes in neuronal firing patterns or the local field potential (defined below). We use the terms acute, concurrent, or online as synonyms of the term “immediate.”

Offline effects—Potential effects of TES which may outlast the period of stimulation.

Local field potential—a composite measure of electric current generated from all active cellular processes within a volume of brain tissue superimpose at a given location in the extracellular medium and generate an extracellular potential *V*_e_ (a scalar measured in Volts) with respect to a reference potential. The difference in *V*_e_ between two locations gives rise to an electric field (a vector, V/m). The local field is monitored by small intracranial electrodes as opposed to those obtained by scalp EEG recordings.

Oscillations—Rhythmic fluctuations in the local field potential, which can range in frequency from ultraslow (0.05 Hz) to ultrafast (500 Hz), with specific oscillation frequencies characterizing particular brain states.

Shunting—During TES application, the flow of current away from the brain due to passage along a low-resistance pathway (skin, subcutaneous tissue), and away from a high resistance pathway (the skull). Current shunting explains why only a small part of the current delivered through TES electrodes actually reaches the brain surface.

## Hypothesized immediate effects of electric fields on neural activity

We define immediate effects of TES as those that occur simultaneously or acutely from the modulation of the membrane potential by the external field. For a sufficiently strong electric field, neuronal firing rates should result from changes in a neuron’s input/output function^19^, facilitated neurotransmitter release from presynaptic terminals^32^, or ectopically induced spikes^33^.

With AC stimulation, current flows from anode to cathode in one half-cycle and reverses direction during the other half-cycle. These half-cycles fields are equal magnitude but opposite direction. Thus, during low-frequency AC stimulation, somata may be alternately depolarized and hyperpolarized, which could mimic effects of anodal and cathodal DC stimulation. If the applied AC frequency is much faster than the neuronal membrane time constant (~30  ms, or > 33.3 Hz), the fast-changing polarities of the stimulation may reduce neuronal polarization^34,35^, and thereby reduce unwanted peripheral nerve stimulation effects (see below).

**Fig. 2:**
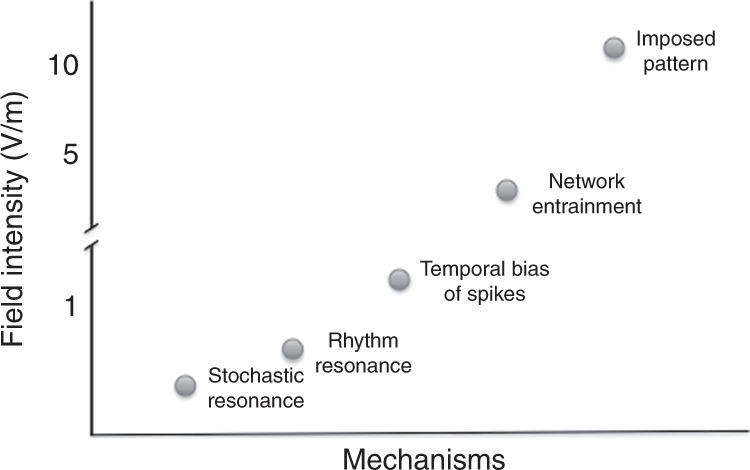
Five postulated mechanisms to affect online spiking of neurons and networks patterns in response to different estimated magnitudes of TES. While the figure illustrates distinct effects, in reality the boundaries of mechanisms are blurred under most experimental conditions. Several mechanisms can act simultaneously in different networks of the same brain. The numbers on the vertical axis are merely estimates based on current data and theoretical considerations

In tACS studies, applied TES current is posited to affect the brain’s native oscillatory patterns^36–38^ and thereby target cognitive and therapeutic applications. While the magnitude of TES-induced physiological effects varies with the strength of the induced field, the ‘effectiveness’ of the stimulation and its consequences on neuronal and network activity depend on many other experimental variables and the brain’s state. We distinguish five neural mechanisms that could affect network activity: stochastic resonance, rhythm resonance, temporal biasing of neuronal spikes, entrainment of network patterns, and imposed patterns (Fig. 2). These five mechanisms of field-induced effects can cooperate or compete with each other and with endogenous brain activity, and can occur simultaneously in different networks of the same brain.

(1) Stochastic resonance. Because physiological (EPSP, IPSP) and exogenous (TES) polarizing mechanisms are added, there is no theoretical ‘minimum effective threshold’ of the induced electric field^39,40^. When a neuron nears the threshold of spike generation, a very small amount of applied field can bias spike timing or spike probability. This is known as ‘stochastic resonance’ ^41^. Weak stochastic effects are hard to quantify in normal networks with fluctuating activity levels because the affected neurons may be scattered in distant networks. Upon repeated application of the field, different neurons may be excited or hyperpolarized, and thus it may be difficult to generate reproducible effects based on this mechanism alone. (2) Rhythm resonance. Under well-controlled, closed-loop conditions, a very weak field can be precisely timed to the depolarizing phase of an oscillating neuron’s membrane potential (see below). Even without a closed-loop system, rhythm resonance may occur when an AC field is applied to a regular endogenous rhythm at the same frequency, because the external field can affect the native oscillation at a similar phase during each cycle^42–50^. Such phase matching may explain tACS beta oscillation phase-dependent modulation of TMS-induced motor responses in the motor cortex^51^. (3) Temporal biasing of spikes. Strong rhythmic applied fields may reliably affect the membrane potential and, consequently, spike timing of subsets of neurons. This mechanism is related to stochastic resonance since the coincidence of intrinsic and extrinsic polarization forces work together but the same neurons are more reliably activated from trial to trial. Such phase-locked temporal biasing of neuronal spikes by the rhythmic forced field may occur without affecting the overall firing rates of neurons. (4) Network entrainment. To entrain network patterns of less regularity (e.g., slow oscillations of sleep) requires stronger currents since exogenous patterns compete with native brain rhythms^19,40,45^. (5) Imposed Pattern. Imposing an arbitrary pattern on a neuronal network (e.g., enforcing theta activity on a network with an endogenous beta rhythm) requires the strongest field to overcome the endogenous control of network neurons (Fig. 2).

Spike and local field potential (LFP) measurements are often-used methods of measuring neural activity in vivo, each with advantages and disadvantages. Spike measures are the most direct read-out of TES effect on neural activity^35^, but are not always available in animals and are rarely possible in humans. LFP is the extracellular measure of transmembrane electric current generated synchronously by a nearby population of neurons, brought about primarily by postsynaptic potentials^52,53^ (see Box 1). Importantly, LFP measurements allow comparison between human and animal experiments. The acute effects of tACS could be measured by changes in the LFP amplitude, such as epileptiform spike-and-waves or the dominant alpha rhythm as a function of the tACS phase^35,54^. A related method is to measure entrainment of slower rhythms by tACS (e.g., sleep slow oscillations, see Box 1), also measured by the amplitude of faster, cross-frequency coupled patterns (e.g., sleep spindles)^17,25^ Quantifying entrainment of rhythms at the same frequency of the applied TES is difficult with current methods due to the difficulty of separating the artifact from the entrained physiological pattern, as discussed in more detail below.

## Acute effects of electric fields in vitro

In brain slices, the effects of induced fields can be studied with exquisite control, since all synaptic signaling can be blocked pharmacologically^55^. Most studies apply constant fields across parallel wires outside the slice. This spatially diffuse stimulation differentially affects the neuronal compartments (e.g., soma, apical dendrites), producing complex effects^23^. In vitro recordings of rat hippocampal and neocortical pyramidal cells show that DC electric fields of 1 V/m can polarize the soma of pyramidal neurons by ~0.2 mV.

**Fig. 3:**
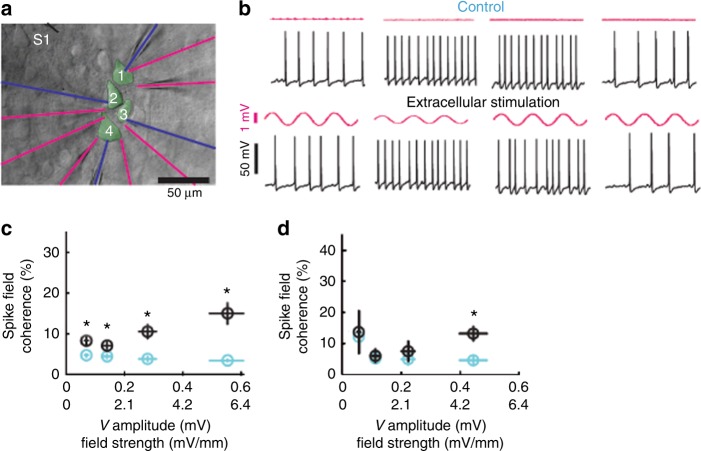
Field-entrainment of spikes under idealized conditions in vitro. Synaptic transmission was blocked pharmacologically. **a** Four neurons with somata located within 100 μm of tissue were patched with intracellular electrodes (blue). Seven extracellular electrodes monitored extracellular voltage (*V*e) fluctuations (magenta). An extracellular stimulation electrode (S1) was placed 50–80 μm from the recorded somata. **b** Each of the four intracellularly recorded neurons were depolarized to induced spiking. Spiking in the absence (top traces) and in the presence of extracellular stimulation (magenta; 100 nA at 1 Hz; bottom traces) is shown. **c** Spike field coherence (circles, mean; error bars, s.e.m.) between spikes and extracellular V*e* is shown during 1 Hz extracellular stimulation (black) and control condition (cyan; circles indicate mean *V*e amplitude at the soma and error bars indicate s.e.m.) as a function of stimulation strength. Asterisks indicate statistical significance of the spike-field difference between control and extracellular stimulation. **d** Spike-spike coordination (spike-field coherence for two simultaneously occurring spikes; essentially spike synchrony among neurons) during 1 Hz extracellular stimulation (black) and control (cyan) condition. Note that stronger fields are needed for coherent entrainment of neuronal spikes across the four neurons (**d**) than for inducing spike-field coherence for each neuron separately (**c**). Figure reproduced with permission^56^

Yet, extracellular electric fields abound in the brain^22^. Local electric fields (> 0.5 V/m) applied in vitro with a micropipette near layer 5 pyramidal cell dendrites and soma induce small intracellular voltage fluctuations (e.g., 0.2 mV) and may phase-lock the spikes to the low-frequency (1–30 Hz) oscillating external field without affecting overall spiking rate^56^. Thus, under idealized conditions, very weak but localized extracellular fields can bias neuronal activity (Fig. 3). Spike timing is highly sensitive to small, persistent oscillations that act throughout a volume. This voltage fluctuation is comparable to intrinsic, non-synaptic, internally generated noise in layer 5 pyramidal neurons (0.2–0.4 mV)^57^ and is much smaller than synaptic background activity in vivo^33,58^. For example, hippocampal theta oscillations, for instance, induce ~4 V/m electric fields across the CA1 pyramidal layer, while hippocampal sharp waves induce 5–15 V/m^39^. Endogenous field strengths associated with slow oscillations in the neocortex are 1–2 V/m^45^. Whether weak applied electric fields can overcome such strong endogenous fields remains an important experimental question.

AC fields of sufficient strength applied at low frequencies (< 10 Hz) can modulate firing rate periodically by the same mechanisms as DC fields^59^. For higher AC stimulation frequencies, the induced effects are diminished; for example, somatic polarization is reduced by ~70% when a 50 Hz AC stimulation induces a 1 V/m field, because the membrane cannot follow the rapid fluctuations in the electric field^60^. The oscillatory fields may not affect the overall firing rate but can modulate the timing of ongoing spikes^56,59^ (Fig. 3). AC stimulation at a frequency matching the resonant neuronal properties can induce cumulative effects over multiple cycles^43,45,59–62^. Under such ideal conditions, AC fields as low as 0.2–0.5 V/m can shift spike timing^23,43,59,60^. Higher stimulation frequencies require stronger currents to bias spike-LFP coupling (> 0.7 V/m induced field at 1 Hz and > 5 V/m at 30 Hz^56,39^, Fig. 3), due to the frequency filtering properties of the neuronal membrane^63^. One caveat of these in vitro studies is that pharmacologically or intracellularly induced oscillations are much more regular than in vivo oscillations^19^. Overall, in vitro studies have laid the biophysical foundation for TES, providing quantitative relationships between the effective magnitude of voltage gradients for both V_m_ and spiking activity. These experiments have also clarified the geometric relationship between the vectorial field and neuronal morphology and soma-dendritic orientation. However, these studies do not reveal how applied current affects neurons when applied through the skin, subcutaneous tissue, skull, dura and cerebrospinal fluid in the living animal. For a detailed summary of in vitro experiments measuring acute physiological changes during TES, see Supplementary Table 1.

## Acute effects of external electric fields in rodents

In rodent experiments, electric fields are often applied with electrodes placed on the skull, the dura mater, or directly on the brain surface. Often, a saline-filled cup is applied at various epicranial locations with a return electrode on the thorax or the contralateral hemisphere. Several in vivo studies have found a dose-dependent relationship between the induced electric field and spiking rate^24,34,35,40^, transmembrane potential and, at higher stimulation intensities, on the LFP^25,35,39^. Applied electric fields can alter neuronal excitability^64^ and evoked responses^64,65^ in a polarity-specific manner.

However, rodent studies typically use ten-fold stronger current intensities compared to human studies^66^. Across the 28 rodent experiments reviewed^67^ (Supplementary Table 2), the induced intracranial fields averaged 6.8 ± 3.8 V/m (*n* = 11, ten epicranial and 1 subdural studies), compared to < 1 V/m measured in conventional human TES studies^19,68,69^. While stimulating transcranially in rats, the lowest electric field sufficient to affect the timing of spiking activity in widespread cortical and hippocampal areas was ~1 V/m; higher intensities were required to reliably affect LFP and the membrane potential in intracellularly recorded neurons in vivo^35,40^. Higher field intensities were also required in urethane-anesthetized rats to affect LFP oscillations^70,71^. Similarly, applied currents have terminated thalamocortical spike and wave patterns in rodents (1.5–2 mA, applied to skull)^54,72^ or triggered paw movement (0.25 mA and 0.9 mA)^34^ and modulated endogenous slow wave initiation and propagation patterns (~3 mA /mm^2^)^73^. In summary, rodent stimulation studies demonstrate physiological effects on spike timing, LFP oscillations, and terminating seizure patterns, but applied currents were several-fold stronger than the currents expected to penetrate the human brain using TES. For a detailed summary of in vivo experiments measuring acute physiological changes during TES, see Supplementary Table 2.

## Immediate electrophysiological effects of TES in primates

Non-human primates permit TES investigations using a relatively large head with a gyrated cortical surface. The variable anatomy, including thick skull and large musculature, can result in a wide range of field strengths across studies and even across individuals. Opitz et al.^69^ measured field strengths in two anesthetized cebus monkeys with small round scalp electrodes (3.14 cm^2^) over the left occipital and frontal cortices stimulated with 1– 2 mA. The median induced electric fields were 0.21 V/m (strongest 0.36 V/m) in a male monkey (4.1 kg) and 0.39 V/m (strongest 1.16 V/m) in a female monkey (2.9 kg). The weaker effects in the male likely reflected the larger head musculature that shunted much of the applied current.

Krause et al.^74^ studied the effects of tDCS with intracranial electrodes in right prefrontal and left inferotemporal cortices in macaque monkeys performing an oculomotor foraging task. Based on a finite-element model^75^, 2 mA anodal stimulation applied to the right prefrontal region induced field strengths between 0.4–0.7 V/m. Although tDCS did not affect the spontaneous or task-related firing rates of isolated single units or multi-unit clusters, LFP power in the 1–15 Hz band increased ten-fold during DC stimulation, and coherence within the prefrontal array increased at all frequencies from 2–100 Hz. However, the changes in spike frequency or spike-field coherence in areas with stronger fields (up to 1 V/m) but outside the recording area, could not be ruled out.

Kar et al.^76^ delivered tACS of 1 mA peak amplitude at 10 Hz frequency through scalp electrodes placed anterior to the vertex and lateral to the middle temporal area (MT). In one monkey, the induced field was 0.12 V/m at closely spaced electrodes (0.5 mm) in the recorded MT area. Spiking activity during the stimulation was not reported, but a reduction in spike adaptation to the visual input after tACS offset was observed.

In a dose-ranging tDCS experiment on motor control, Lee et al^77^. applied anodal stimulation over primary motor cortex (with cathode at the vertex) to determine the threshold to elicit a muscle twitch in sedated macaques. Between 50–120 mA (~35–55 V/m electric fields in the motor cortex) were needed to trigger a visible twitch.

In summary, primate models offer the advantage of testing TES in a larger head model with a gyrated cortical surface. The few primate studies which have been performed to date employ different montages and measurement techniques. Compared to the rodent models, primate experiments are limited by weaker induced fields and proportionately less cortical recording coverage. Primates with large heads, thick skulls, and greater musculature have greater shunting, or current loss away from the brain, and require larger current intensities to achieve comparable electric field strengths to those in rodent brains. These differences highlight the critical need to match intracerebral fields rather than stimulation parameters across species and experiments.

## Ex vivo human studies

**Fig. 4:**
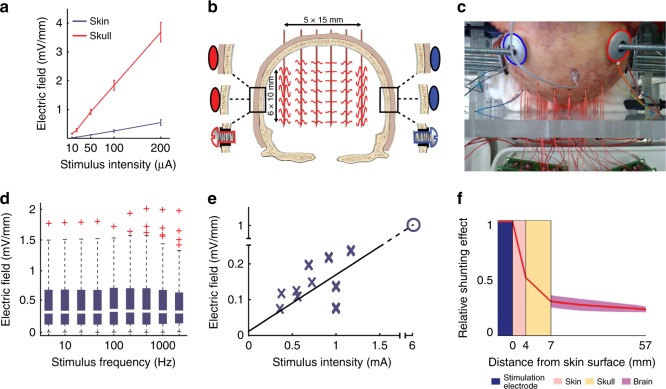
Current loss of TES in rodents and in human cadaver brains. **a** Compared to subcutaneous stimulation (red), transcutaneous stimulation (blue) generated several-fold weaker electric fields in a rodent model. **b** Schematic of the experimental arrangement for transcutaneous, subcutaneous, and epidural stimulation in cadavers, in a coronal plane. **c** Photograph of the cadaver skull with drilled holes and inserted matrix of recording electrodes. Stimulation electrodes, marked by blue and red circles for negative and positive polarity, respectively, were fixed to the skull. **d** Effect of stimulus frequency on intracerebral voltage gradients. Stimulus frequency between 5 and 1000 Hz has a minor effect on intracerebral gradients. **e** Extrapolation from measurements in human cadavers (blue crosses) suggests that approximately 6 mA current applied across the skin would induce 1 V/m intracerebral electric field (blue open circle). **f** Attenuation of charge flow (red line) through scalp (pink), skull (yellow), and brain (dark pink), as measured in human cadaver heads. Figure adapted from Figs. 1, 4, 5 in Voroslakos et al., (2018)^35^, under the Creative Commons License

Translating the results of in vitro and in vivo animal experiments to humans is confounded by complexities of brain and head anatomy. Neurons in highly gyrated cortices of higher primates have variable alignments even on the convexity^29,78^, in contrast to those of lissencephalic rodents which are mostly perpendicular to the skull surface. Since entrainment efficacy of neurons depends on how polarized neurons align with the direction of the electrical field, TES of human cortical gyri may lead to unpredictable effects. Further, safety considerations require TES application through the hair, skin, and skull. In contrast, few animal experiments applied TES through the scalp^35,69,74,76^. Using transcranially applied stimulation, only a small fraction of the applied current enters the brain: most is lost through the skin, subcutaneous soft tissue, and the skull^35^ (Fig. 4a). One study conducted in rodents and human cadavers demonstrated that > 75% of applied TES current is lost through shunting^35^, although the shunting effects differ between humans, nonhuman primates and rodents in unknown ways.

Human cadavers yield insights regarding conductivity and anisotropy. Multiple intraparenchymal, three-dimensional measurements of the electric field distribution can be obtained and multiple stimulation parameters can be tested in the same cadaver (Fig. 4b, c). Such empirical measurements help determine the basic biophysical tissue properties and help model effects of TES into the brain parenchyma^79–81^ (Fig. 4d–f). Intracerebral electric fields were measured in intact cadaver brains at 198 sites in a three-dimensional grid, while applying stimuli that varied in frequency, intensity, phase relationship, electrode location, and size^35^. The authors concluded that scalp, skull, brain, and cerebrospinal fluid behave as ohmic conductors. In other words, induced fields increased linearly with stimulation intensity, but were independent of stimulation frequency^82^. The comparison of transcutaneous, subcutaneous and epidural stimulations of the cadaver brain led to the conclusion that the bilateral transcutaneous stimulation of the cadaver heads with traditional montages and intensity (peak 1 mA) induced only ~0.2 V/m field in the brain^35^. Of note, death imparts profound changes in the biophysical properties of brain tissue, limiting a direct ex vivo to in vivo comparison^83^.

## In vivo human studies

A critical question is the magnitude of the electric field reaching the brain when using conventional TES, applied transcutaneously at peak intensity currents of 1–2 mA, because field strength constrains the expected nature of the physiological changes (Fig. 2). The main barrier for TES to reach the brain is the skull’s high resistance (~160 Ωm) combined with the low resistance of the scalp (~2 Ωm)^80^. A large fraction (up to 75%) of the current seeks the path of least resistance and shunts across the scalp^35^. Three independent studies using intracranial electrodes in epilepsy patients undergoing surgical evaluation demonstrated that maximal electric fields at the cortical surface under the electrodes were < 0.5 V/m for 1 mA peak intensity currents^68,69,84^.

Can such weak electric fields reaching the human brain induce changes in neuronal networks, as measured by changes of the LFP? Oscillations are ubiquitous in the human brain, ranging from ultraslow (0.05 Hz) to ultrafast (500 Hz), with specific oscillation frequencies characterizing different arousal states or cognitive processes and coordinating activity between local and distant brain regions^85^. A leading hypothesis about the behavioral effect of tACS is that the applied stimulation frequency matching the dominant network oscillation entrains and augments endogenous rhythms^37,38,62,86–89^, such as gamma^60,89,90^, beta^91^, alpha^46^, theta^92^, and slow frequency oscillations^40,45,93^.

Whether the weak electric fields resulting from TES at conventional intensities (1–2 mA peak) can entrain neuronal oscillations is technically difficult to assess. One technical challenge is that the magnitude of the stimulation artifact is dramatically larger than the magnitude of the endogenous oscillation, making artifact rejection difficult. To illustrate, supposing the use of a 10 Hz tACS waveform to entrain endogenous alpha oscillations, the amplitude of alpha waves is ~ 100 µV over the parietal-occipital lobes, whereas the artifact induced by a 2 mA tACS stimulus can exceed 1 V (~1000x greater). Therefore, even if 99.9% of the artifact is removed, the remaining 0.1% of the artifact would be as large as the endogenous alpha rhythm. Further, the stimulus waveform can be distorted, resulting in multiple harmonics of the applied signal. Due to non-linearities and instability of the stimulation waveform during the experiment, the waveform of the stimulation artifact and its harmonics across trials cannot simply be subtracted from the resulting EEG recordings. While artifact rejection algorithms can clean the recorded signals, artifact-free brain activity is unlikely to be recovered when the stimulation frequency matches the endogenous oscillation frequency^94^. Another challenge is the saturation of recording amplifiers, which precludes analysis of the endogenous LFP during stimulation.

Besides neurophysiology, other experimental measures of TES immediate effects include blinded subject reporting, TMS-evoked motor evoked potential (MEP), and fMRI. Feurra et al.^49^ applied at 1.5 mA anodal current at varying frequencies of (2–70 Hz) over somatosensory cortex and asked subjects to report tactile sensation in the contralateral hand. While no sham or control stimulation site was included, during one of the two replications, subjects reliably reported sensation during alpha (10–14 Hz) and high gamma (52–70 Hz) frequency stimulation. These occasional behavioral effects of TES may reflect stochastic resonance (Fig. 3).

A study of TMS-evoked MEPs during tACS (1 mA peak-to-peak) at different frequencies (theta, alpha, beta, and gamma) over the primary motor cortex during rest and during a motor imagery task found MEPs were greatest during beta frequency tACS at rest, but during theta–tACS during motor imagery^38^. Guerra et al.^51^, found beta frequency (1 mA peak-to-peak, 20 Hz) tACS stimulation acutely modulated the MEP amplitude in a phase-dependent manner; however another experiment^37^ (1.5 mA peak-to-peak) did not find phase-dependent modulation of corticospinal responses using low-frequency tACS (0.8 Hz) or tDCS superimposed with 0.8 Hz. An fMRI study applied 20 Hz tACS stimulation (1 mA peak-to-peak) over the primary motor cortex (M1) found enhanced local connectivity within M1, without changing the overall local activity or long-range connectivity within the default mode network^95^. While these TMS and fMRI findings are consistent with a rhythm-resonance mechanism (i.e., 20 Hz modulation of resting beta frequency in motor cortex; Fig. 2), a peripheral contribution remains possible. Both consciously detected and subthreshold (unconscious) sensory stimuli can influence neocortical activity^96,97^.

Epilepsy patients undergoing invasive monitoring offer the opportunity to measure and analyze endogenous oscillations during TES and thus directly monitor brain responses, since intracranial signals are an order of magnitude larger than those recorded from the scalp. The effects of tACS (0.75 Hz and 1 Hz) during NREM sleep and waking rest were assessed in epilepsy surgery patients at standard stimulation intensities (up to 2.5 mA peak to peak; maximum induced field: 0.43 V/m). During NREM sleep, slow oscillations (~1 Hz) strongly entrain spindle activity in the 10–15 Hz range. This provides the opportunity to test whether endogenous slow-wave rhythms can be entrained with low frequency (0.75 Hz or 1 Hz) tACS, while measuring entrainment in a different frequency band that is largely uncontaminated by stimulation artifacts. While spindle and gamma activity robustly entrained to the phase of the endogenous slow oscillation, low-frequency tACS failed to entrain spindle or gamma activity at over 1000 electrode sites measured in patients during NREM sleep. Likewise, no entrainment of gamma or theta activity was observed during waking rest^19,98^.

Instead of entrainment, TES-phase/EEG-amplitude coupling can also be examined with scalp recordings. This approach was validated by concurrent unit recordings in rat experiments^54^. In agreement with the intracerebral recordings^19^, no detectable effect was obtained on the scalp EEG when TES was applied at < 2.5 mA in healthy subjects^35^. However, reliable tACS phase modulation of the amplitude of alpha rhythms was present when intensities exceeded 4.5–6 mA. The effect was specific for neuronal stimulation of cortical neurons because alpha amplitude enhancement over the left and right occipital cortex occurred in phase with alternating the anodal stimulation. Furthermore, to rule out peripheral contributions, control experiments applying stimulation to the abdomen did not produce an effect on alpha activity.

## Novel TES methods

Current applied through the scalp stimulates electrically sensitive elements from the scalp surface to the brain. These peripheral side effects, including stimulation of cranial nerves, retina, and vestibular system, limit the maximum tolerable dosing in humans using traditional TES approaches. Currents exceeding 1–2 mA (depending on electrode type) can cause itching, burning sensation, pain at the skin under and around the electrodes^99^.

The next generation of TES technologies should make three advancements: (1) delivery of stronger currents to the brain while minimizing peripheral and indirect effects; (2) simultaneous stimulation and recording of brain activity for quantitative measurements of TES-induced effects; and (3) targeting of specific rhythms through closed-loop stimulation of brain areas, including deep brain structures.

Multi-electrode stimulation is one potential method to increase current density and focality of stimulation delivered to the brain surface. The strongest transmembrane polarization is expected to build up where electric fields overlap inside the head, whereas the intensity of local scalp stimulation is divided proportionally by the number of stimulation electrode pairs. The approach is similar to previous efforts which used multiple small electrodes to optimally target specific brain areas^100^.

Two other approaches attempt to increase the brain to scalp stimulation ratio. The first is temporal interference stimulation. First introduced for stimulating peripheral nerves and brain^101^, temporal interference is now routinely used in physical therapy^102^. When applied to the mouse brain, intersecting 2 kHz and 2.01 kHz sinusoidal waveforms yielded a 10 Hz amplitude modulation of the LFP^34^. Because the high-frequency stimulus exceeds the time constant of the peripheral nerve membrane, the fast alternating current may reduce skin sensations and avoid retinal responses, while the interfering signals in the brain are expected to be strong enough to modulate neuronal firing. The second approach, **‘**intersectional short pulse‘ (or ISP) stimulation, uses multiple electrode pairs^35^. This time-multiplexing method exploits the time integrating property of the neuronal membrane (i.e., the membrane time constant of cortical neurons is ~30 ms), by applying currents that switch every 60 µs between the electrode pairs. The strongest transmembrane charge will build up where successively induced electric fields overlap inside the head, whereas the intensity of local scalp stimulation is divided proportionally by the number of stimulation electrode pairs. Again, the more electrode pairs are used, the greater the potential to generate brain focal stimulation while minimizing stimulation of the scalp to reduce skin sensations^78,100^. Initial studies using temporal interference and ISP show increased spatial targeting in animal models, but further analysis and refinements of these techniques are necessary.

The application of high-frequency pulses avoids the saturation of recording amplifiers and allows for the simultaneous measurement of the direct physiological effects of tES in humans^35^. Amplifier modifications, such as front-end subtraction of the applied tES waveform can further reduce induced artifacts. Such innovations are essential to directly quantify the physiological responses of neuronal circuits.

## Recommendations for future experiments

Although the lasting effects of stimulation are the main purpose of most clinical trials^103,104^, our review focused on the immediate physiological effects of TES on spikes and network dynamics. A more accurate and sophisticated understanding of physiology, grounded in empirical measurements, will improve design and execution of future TES experiments, with more potent and consistent clinical benefits. Below, we offer a list of tasks for future TES research.

### Exploration of peripheral and non-neuronal mechanisms of TES

It is tacitly or explicitly assumed that behavioral and clinical effects of TES are mediated by directly affecting neuronal activity in the brain. However, behavioral and cognitive changes in response to scalp electric stimulation may be mediated by other effects as well. Electric fields affect the excitability of subcutaneous nerves which signal to the brain. Even when the subject does not report a conscious experience of TES, sensory stimulation can still indirectly affect brain circuits^97,105^. Therefore, control experiments that measure the conscious and subconscious effects of sensory stimulation should be included in future studies. This can be achieved by including an “active control,” such as stimulation of the neck or abdominal areas^35^ or application of TES to another region of the brain^106^. In addition, applying topical anesthesia to the scalp under the electrodes and testing varying intensities of skin stimulation could exclude or characterize sensory contributions.

Long-lasting TES effects can be indirectly mediated by non-neuronal mechanisms as well, including trophic factors^107^, neurotransmitter metabolism^51,108^ glia^109,110^, neuroendocrine system^111^, fibroblasts^112^ lymphocytes, and other electrically charged immune system components^113^. These potential non-neuronal effects should be considered with low-intensity scalp stimulation, which may be too weak to induce the necessary electric field strengths to instantaneously affect neuronal activity. These indirect effects may play a role even when neuronal activity is directly affected by stronger TES.

### Targeting neural circuits

Growing evidence, summarized above, suggests that TES applied at ± 1 mA peak intensity (2 mA peak to peak) generates < 0.5 V/m electric fields in the human brain. This is sufficient to generate 0.1–0.2 mV changes in the membrane potential of cells within the stimulated area. As these changes are significantly lower than the 20 mV depolarization required to bring a neuron from its resting potential to spike threshold in vitro, TES is unlikely to directly elicit changes in spiking activity. Instead, weak induced fields may be more effective when used to bias or augment ongoing rhythms instead of introducing new activity patterns. Applying current at the optimal phase of endogenous rhythms in a closed-loop system may be most effective^54^. However, such responsive methods require characterization of the affected circuitry and continuous monitoring and adjustment of the relevant rhythms^114^. This may be achieved with weak electric fields but other applications may require higher field strengths. For example, prompt control of spiking activity (e.g., to terminate a seizure) may require field strengths > 5 V/m^72^.

### Modeling for spatial targeting and to increase replication across subjects and studies

**Fig. 5:**
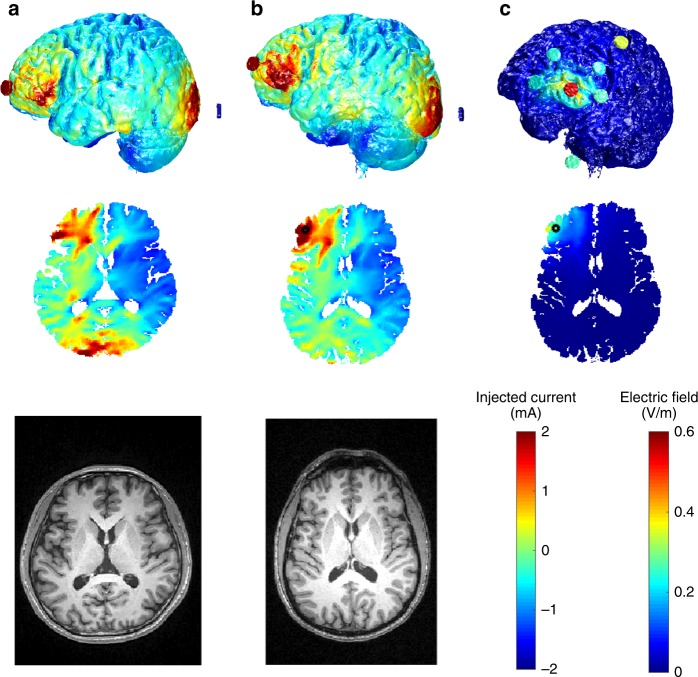
Individualized models of transcranial electrical stimulation for two subjects with variable electrode placements. **a** Model of Subject 1, with 2 mA current injected at electrode Fp1 and return from electrode P3. **b** Model of Subject 2 with the same electrode configuration. **c** Model of Subject 2 with another electrode configuration attempting to achieve more focal stimulation. Note that stimulation is more focal but achieves weaker fields compared to **b**, due to increased current shunting through the skin between near-by electrodes. Electrode montages in **b** and **c** are obtained by a numerical optimization algorithm that attempts to achieve maximal intensity (**b**) or maximal focality (**c**) for the location indicated by a black circle. The current-flow models were generated from previously published data^118^ and methods^119^. We used ROAST, a toolbox for realistic current-flow models of the human^119^

Individual variations in neuroanatomy (scalp, muscle, and skull thickness) can significantly alter the strength and distribution of the induced electrical field in humans^68,98,115–117^ and experimental animals^75,77^, and thus may account for inconsistent behavioral results in human TES experiments. This is exemplified by Fig. 5, which compares two individual models, with the same surface electrode montage but different head anatomy (Fig. 5a, b, created using previously published data and methods^118,119^). Differences in skull thickness and cerebrospinal fluid volume in posterior regions lead to different field distributions (Fig. 5a, b).

Computational models of current flow have been continuously refined over the last decade^78,100,116,117,120–122^. Recent head models can improve spatial targeting by guiding stimulation electrode placement (Fig. 5c). These models are based on segmentation of tissue (at 1 mm^3^ resolution) into compartments of differing electrical conductivities, including skin and other soft tissues, skull, gray matter, white matter, ventricles, and cerebrospinal fluid. Models indicate that focal stimulation of a few centimeters may be achieved at the cortical surface^100^, generally with a trade-off between focality and intensity of stimulation (Fig. 5). Deep targets may be reached at relatively high field intensities when adjacent to CSF-filled ventricles, which can guide currents deep into the brain^68^.

Computational models can help optimize electrode locations for maximal stimulation of a desired brain target^100^. Individual MRI head anatomy combined with toolboxes such as ROAST, and SimNIBS, and SCIrun can define field distribution for this purpose. When an individual’s head MRI is not available, validated and calibrated universal head models can help reproduce in vivo intracranial measurements^118,119,123^. Furthermore, multielectrode montages can be optimized to engage local and network level targets, as demonstrated by a technique combining electric field modeling and cortical networks derived from PET or resting state functional connectivity MRI^124^.

Human TES experiments typically report the stimulation protocols by reporting the stimulating electrode positions (e.g., “anode at F8” or “over left dorsolateral PFC”) and current intensities (e.g., “2 mA”). These specifications describe how the experiment was designed and performed, but they only indirectly define the induced electrical field strength and distribution. Reporting induced field estimates from head models would help relate behavioral and clinical reports to underlying neural mechanisms. When the individual MRI head is available, subject-specific estimates of the induced field strength should be included as a covariate in analyses of its behavioral or neural effects. Intensity-response curves, inverted polarity stimulations and skin stimulations far from the desired target location may distinguish between direct brain-stimulation and peripheral effects. Measurements or estimates of the electric field strength are included in some recent human and large animal studies but are missing from most rodent studies. Reporting field magnitudes is important for animal studies where variable head size and anatomy can strongly affect field magnitudes. These values would provide a critical translational scaling metric to facilitate comparisons with human and animal studies and should be included in future publications.

### Hardware and signal processing issues

The simultaneous measurement of physiological activity (EEG, ECoG) during tACS is complicated by stimulation artifacts several orders of magnitude larger than intrinsic brain activity (1–100 µV). When brain activity and stimulation are recorded simultaneously, special care should be taken to (1) avoid saturation of the recording amplifier during experiments, and (2) subtract stimulation artifacts from the measured signal. Saturation can be avoided by using high-quality amplifiers with sufficient input dynamic range (e.g. an amplifier with dynamic range of ± 100 mV can capture the full stimulation artifact).

Artifact rejection methods initially suggested for scalp EEG^87^ are highly unlikely to fully recover artifact-free brain activity (see above)^94,125^. Therefore, when using tACS stimulation, it is difficult to analyze online neural responses at the same frequency in which stimulation is applied (e.g., increases in alpha oscillations during 10 Hz tACS stimulation). Analysis should target frequencies well away from the stimulation frequency and its harmonics. Measurements using phase-amplitude coupling or single-unit recordings may also be used when available.

DC stimulation artifacts pose less of an issue during signal processing as hardware filters will typically remove any DC offset (avoiding amplifier saturation). However, EEG recordings in humans are affected by artifacts in the 1–15 Hz frequency band due to heartbeats and eye-blink^125,126^. The effect is most prominent in frontal locations. In addition, care should be taken to avoid motion of the subject and stimulation electrodes, electrical noise injected into the system is reduced by using high-quality (i.e., narrow band) stimulation devices, and environmental noise (ensure adequate electrical isolation^19,68^. Control experiments, e.g., using a phantom head model, are needed to separate TES-related artifacts from the induced neural activity.

### Data sharing standards

Because different devices use different conventions, it is critical to specify whether tACS amplitudes describing the absolute magnitude (i.e., the amplitude of one phase), the peak-to-peak amplitude, or another form of parameterization. Shared data and analysis methods can facilitate coordinated community efforts and metanalysis. Combining many data sets may offer novel insights and interpretations (e.g., https://www.nwb.org/). Further, given the artifact rejection methods described above, sharing raw EEG data sets would enhance replication of the signal processing pipeline. Sharing analysis software packages will facilitate cross-validation of results across studies.

## Conclusions

A more accurate and sophisticated understanding of TES physiology, grounded in empirical measurements, will improve the design and execution of neurostimulation experiments, yielding more robust and consistent clinical benefits. While the authors of this review share a consensus on experimental recommendations, we acknowledge a controversy within the TES field and amongst ourselves as to whether the attenuated electrical fields that reach the human brain with conventional protocols can directly affect neurons and neuronal networks. While direct neural mechanisms have been measured with exquisite experimental control in vitro and in vivo models, whether these insights translate to humans is uncertain. We have highlighted challenges in translating mechanistic insights from experimental models to humans, including (1) the interaction of the applied external field with competing or cooperating network oscillations when comparing in vitro to in vivo studies; (2) external fields applied to the folded cortical surface produce more variable and unpredictable effects in larger animals (including humans) compared to rodent and in vitro models; (3) the large human head and the low resistance scalp shunt most applied current, with much smaller fraction of the applied current reaching the cortical surface.

The weak induced electric fields reaching the human brain contrast with the numerous behavioral and clinical effects reported. We should also consider how TES can affect brain activity indirectly, including activation of afferent nerves^127^, retina and the vestibular apparatus^42^, astrocytes, perivascular elements, and glial activation^109,110^, as well as through placebo effects^128^. Both tACS and tDCS may also induce cumulative or longer-term effects via unknown mechanisms that merit further exploration. Future human behavioral trials should control for sensory contributions with approaches such as topical anesthesia, or by using sensory controls such as stimulation away from the desired targets.

Novel stimulation methods that safely induce higher brain to scalp electric field ratios are needed. Existing experimental techniques, such as temporal interference stimulation and intersectional short pulse stimulation delivering stimulation at high frequencies (> 1 kHz), require further testing in both experimental animals and humans. These and related methods, such as multielectrode TES^78,100^, may better target brain structures compared to conventional electrode montages. Closed-loop techniques may enhance the efficacy of TES via resonant modulation of native brain rhythms. In selected cases, supported by experimental findings and human head-based modeling, subcutaneous arrays or electrode plates inserted below the skin may provide superior efficacy, similar to techniques to treat headache^129^ and facial pain^129,130^ by stimulating peripheral nerves. With further development, TES could become a leading tool for chronic, on-demand, at-home treatment of many neuropsychiatric and neurological diseases.

## Electronic supplementary material

Supplementary Information
