# Supplementary material for: Immediate neurophysiological effects of transcranial electrical stimulation
Source: Nat Commun. 2018 Nov 30;9:5092. doi: 10.1038/s41467-018-07233-7 (PMC6269428; doi:10.1038/s41467-018-07233-7)
Supplement: Supplementary file 1 — Supplementary Information [file 41467_2018_7233_MOESM1_ESM.docx]

**Supplementary Table 1. Summary of *in vitro* experiments measuring acute physiological changes to TES**

| **Study** | **Measured Effects** | **Main findings** | **Brain Region/**  **Cell type** | **Stimulation Waveform/**  **Frequency** | **Peak Electric Field** | **Stimulation**  **Timing** | **Recording**  **Methods** |
| --- | --- | --- | --- | --- | --- | --- | --- |
| (Reato, Rahman et al. 2010) | -Spike Entrainment (0.2 V/m, 26 Hz) -Firing Rate (6 V/m, DC) -Gamma LFP Power (6 V/m, DC; 4 V/m, 2 Hz) | -6 V/m DC modulated gamma power and firing rate  -AC stimulation up to 12 Hz is more effective than DC at modulating gamma power, but no change in firing rate overall ->4 V/m AC stimulation at higher frequency (28 Hz) leads to subharmonics, and reduction in power at the endogenous frequency.  -0.2 V/m entrains single units when stimulation is matched to endogenous oscillation frequency | -Rat hippocampus CA3 | 0-26 Hz | 0.2-10 V/m | -Carbachol induced gamma oscillations (25-35 Hz) | -Sharp intracellular and extracellular recordings |
| (Francis, Gluckman et al. 2003) | -Population Burst Entrainment (0.3 V/m, 2 Hz) -Spike Entrainment (0.4 V/m, 2 Hz) | - CA1 Bursts are entrained as low as .295 V/m (peak amplitude) -Higher threshold for entrainment of CA1 units (0.393 V/m) and CA3 bursts (0.787 V/m) | -Rat hippocampus CA1 and CA3 | -Gaussian pulse (26 ms width at half-max) -1 to 2 Hz | 0.295 V/m | -High potassium induced complex bursts | -Extracellular and single unit recordings |
| (Deans, Powell et al. 2007) | -Spike Entrainment (0.5 V/m, 50 Hz) -Gamma LFP Power (0.5 V/m, 50 Hz) | -Polarization drops off exponentially with increasing stimulation frequency (resonance around 10 Hz)  -50 Hz AC modulates gamma power and peak frequency -0.5 V/m at 50 Hz entrains single units | -Rat hippocampus CA3 | 20-50 Hz | 0.5-10 V/m | -Kainic acid induced gamma oscillations (30 Hz) | -Sharp intracellular and extracellular recordings |
| (Anastassiou, Perin et al. 2011) | -Spike Entrainment (0.7 V/m, 1 Hz; 1.5 V/m, 8 Hz; 5.9 V/m, 30 Hz) | -Spikes entrain to AC field >0.7 V/m (more effective at lower frequencies) -Electric field is nonuniform (generated by point source). Stronger uniform fields are likely needed -Multiple neurons entrain simulatneously | -Rat somatosensory cortex layer V pyramidal neurons | 1, 8, 30 Hz | 0.7, 1.5, 3.0, 5.9 V/m (nonuniform, from point source) | -Intracellular current injection for regular firing | -Whole cell and extracellular recording |
| (Frohlich and McCormick 2010) | -Slow Oscillation Entrainment (1 V/m, 0.1 Hz) | -Slow oscillation upstates are entrained by fields as low as 1 V/m -Entrainment is more effective the closer the stimulation frequency is to the endogenous frequency -Slow oscillation entrains to 0.5 V/m when waveform is based on natural oscillations recorded in vivo | -Ferret visual cortex | 0.1-1 Hz (matched to endogenous frequency, closed loop) | 0.5-4 V/m | -Spontaneous slow oscillations in infragranular layer | -Sharp intracellular, extracellular recording array |
| (Terzuolo and Bullock 1956) | -Firing rate (1 V/m, DC) | -1 V/m required for change in spontaneous firing rate -Fields along the main axis of the cell were most effective, orthogonal fields ineffective | -Crayfish abdominal stretch receptor -Lobster cardiac ganglion | DC | 1 V/m | -Spontaneous firing in individual cells or bursts in ganglion | -Extracellular electrode recording spontaneous firing and electric field magnitude |
| (Ghai, Bikson et al. 2000) | -Population Burst Suppression (1 V/m, DC) | -Number of bursts decreased monotonically with electric field magnitude -Probability of completely blocking bursts increased with electric field magnitude ->6 V/m: 100 % block, 2 V/m: 50% block,1 V/m: 10% block | -Rat hippocampus CA1 | DC | 1-8 V/m | -Low-calcium bursts | -Extracellular recordings |
| (Radman, Su et al. 2007) | -Spike Timing (1 V/m DC; 1 V/m 30 Hz) | -1 V/m DC alters spike timing in response to current injection -1 V/m at 30 Hz entrains spikes during current injection | -Rat hippocampus CA1 | DC, 30 Hz | 1 V/m | -Intracellular current ramp | -Sharp intracellular recordings |
| (Maeda, Maruyama et al. 2015) | -Spike Entrainment (1.3 V/m, 4 Hz) | -Entrainment of calcium transients in distal apical dendrites as low as 1.3 V/m | -Rat hippocampus CA1 | 1- 4 Hz | 1-20 V/m | -Spontaneous calcium transients in elevated potassium and 4-AP | -Multi-cell calcium imaging with OGB-AM and confocal microscopy |
| (Gluckman, Netoff et al. 1996) | ,-Population Burst Entrainment (2.5 V/m, 3.3 Hz) | -Bursts entrain to 2.5 V/m, 3.3 Hz AC stimulation with noise -With increased noise amplitude, entrainment is enhanced. Peak occurs around 10 V/m noise (RMS) for a 2.5 V/m sinusoid | -Rat hippocampus CA1 | 3.3 Hz + Gaussian noise | 2.5-15 V/m AC; 5-25 V/m noise | -High potassium induced complex bursts | -Extracellullar recordings |
| (Xu, Wolff et al. 2014) | -Spike Entrainment (4 V/m, 1 Hz) | -4 V/m at 1 Hz generates AMPA and NMDAR-dependent voltage transient that entrain to the field | -Mouse barrel cortex | 1-4 Hz | 2-25 V/m | -No reported endogenous activity | -Voltage sensitive dye (NK3630), pharmacology (CNQX, APV) |
| (Jefferys 1981) | -Population Spike (5 V/m, DC) | -DC stimulation modulates evoked population spike amplitude and latency -Dose-response was approximately linear, with effects resolved as low as 5 V/m | -Guinea pig hippocampus granule cell layer | DC | 1-70 v/m | -Evoked synaptic responses  -Varied timing of evoked response relative to field onset (100 or 10 ms delay) | -Extracellular recording electrode in granule cell layer -Population spikes evoked by afferent stimulation of perforant path |
| (Gluckman, Neel et al. 1996) | -Population Burst Suppression (10 V/m, DC) | -Seizure-like bursts propagate from CA3 to CA1 -10 V/m field abolishes seizure events in most slices | -Rat hippocampus CA1 | DC | 10 V/m | -High potassium induced complex bursts | -Extracellular recordings |
| (Bikson, Inoue et al. 2004) | -Population Spike (10 V/m, DC) | -10 V/m modulates population spike amplitude and latency -Fields >100V/m could induce epileptiform activity | -Rat hippocampus CA1 | DC | 10-200 V/m | -Evoked fEPSPs and antidromic population spikes | -Extracellular and sharp intracellular recordings |
| (Reato, Bikson et al. 2015) | -Firing Rate (10 V/m, DC) -Gamma LFP Power (10 V/m, DC) | -10 V/m DC during modulates gamma power and multi-unit firing rate, which lasts for ~10 minutes after stimulation ends | -Rat hippocampus CA3 | DC | 10 V/m | -Carbachol induced gamma oscillations | -Extracellular field potential -Multi-unit activity |
| (Chan and Nicholson 1986) | -Firing rate (15 V/m, 0.05 Hz) | -15 V/m required for detectable effects on spontaneous firing rate in Perkinje cells -Effects on stellate cells were more variable due to differences in geometry | -Turtle cerebellum Purkinje cells and stellate interneurons | 0.05 to 1 Hz | 13-91 V/m | -Spontaneous firing and evoked field potentials | -Extracellular units and evoked (parallel fiber) responses in Purkinje cell soma |
| (Lafon, Rahman et al. 2017) | -Population Spike (35 V/m, DC) | -35 V/m DC modulates fEPSP, population spike, and input/output curve | -Rat hippocampus CA1 | DC | 35 V/m | -Evoked fEPSP and population spike | -Extracellular recordings in CA1 -Stimulation of Schaffer collateral afferents |

**Supplementary Table 2. Summary of *in vivo* experiments measuring acute physiological changes to TES**

| **Study** | **Measured Effects** | **Main findings** | **Species, Age/Wt** | **Brain Region/**  **Cell type** | **Freq** | **Electric Field** | **Stim Electrode Montage** | **Stim Electrode Properties** | **Awake** | **Anaes-**  **thesia** | **Stim Timing** | **Recording Methods** |
| --- | --- | --- | --- | --- | --- | --- | --- | --- | --- | --- | --- | --- |
| (Kar, Duijnhouwer et al. 2017) | -Firing Rate Adaptation (0.12 V/m*, 10 Hz) | -10 Hz tACS reduces adaptation of tuning curves (motion direction vs firing rate) to visual motion ~500 ms after stimulation offset | -2 Adult Rhesus Macaques | MT | 10 Hz | 0.12 V/m | -Transcutaneous -4 cm anterior to vertex on scalp -Return on ear | Reusable surface electrode with viscous mixture: water, isopropanol, aluminum chlorohydrate | X |  | -Awake, duration adaptation to visual stimulus motion | -Tungsten electrodes for extracellular recording |
| (Krause, Zanos et al. 2017) | -Broadband LFP power (0.4 V/m**, DC) -Inter-area coherence (0.4 V/m**, DC) | -DC stimulation targeting PFC led to broadband power increase in PFC only -No effects on firing rate or multi-unit activity -LFP-LFP coherence between PFC and ITC is reduced in delta, theta, alpha bands -LFP-MUA coherence between PFC and ITC is reduced in delta and theta bands | -2 Adult Male Rhesus Macaques | -Prefrontal cortex -Inferotemporal cortex | DC | 0.7, 0.4 V/m** **FEM model | -Transcutaneous (in 10-20 human EEG): Monkey 1: Fp2 +2 mA, O2 -2 mA Monkey 2: F4 +2 mA, P7 -2 mA | Ag/AgCl disk with conductive gel | X |  | -Passive fixation -Visual foraging task | -Multi-electrode utah array in PFC and ITC |
| (Ozen, Sirota et al. 2010) | -Spike Entrainment (1 V/m, 1 Hz) | -Cortical layer 5 neurons entrain to 0.8 - 1.7 Hz tACS -Number of entrained units scaled with stimulation intensity -Anesthesia slow waves: 6% of units entrained to 0.3 V/m, 20% entrain to 1 V/m -Natural slow waves: 25-50% of units entrained to 1 V/m -Exploratory theta oscillations: no entrainment regardless of intensity (mismatch between stimulation and endogenous frequency) | -Male Sprague Dawley Rats, 100-400 g (acute experiments) -Male Long Evans Rats, 250-350 g (chronic experiments) | -Left medial prefrontal cortex -Somatosensory cortex -Hippocampus | ~1 Hz | 0.25-3 V/m | -Epicranial -One electrode on calvarium midline above olfactory bulb -Two electrodes Bilaterally on temporal bone ~6 mm posterior from midline electrode | Stainless steel wire wrapped in spiral | X | X | -Anesthesia induced slow waves -Natural sleep slow waves  -Free exploration theta oscillations | -Silicon probes for LFP recording -Intracellular recording in somatosensory cortex |
| (Voroslakos, Takeuchi et al. 2018) | -Firing Rate (1 V/m, DC, ISP) -Delta LFP Power (9 V/m, DC) | -1 V/m required to detect change in firing rate.  - >9 V/m modulates delta band power -Intersectional pulsed stimulation (ISP) can preferentially modulate firing rate on one side of the brain | -16 female, 3 male Long Evans rats, 350-450 g -8 male Wistar rats, 250-450 g | Visual cortex, Hippocampus | DC, ISP | -3, 6, 9, 12 V/m subcutaneous -0.4, 0.8, 1.2, 1.6 V/m transcutaneous | -Epicranial, bilaterally on temporal bone -Transcutaneous on shaved scalp | Silicon pockets filled with conductive gel |  | X | -Anesthesia | -Intracellular and local field potential recording -ISP: 2.5 or 10 us DC pulses, 5 or 50 us pause, switching between electrode pairs |
| (Berenyi et al. 2012) | -Spike Entrainment (1 V/m, 1 Hz) -Spike-wave episode disruption (10 V/m, closed loop) | -1 Hz tACS entrains multi-unit activity and the spike component of spontaneous spike-wave discharges -Gaussian pulse stimulation (10 V/m, 50 ms) in response to spike-wave onset reduced duration of spike-wave episodes | -Male Long Evans Rats, 510-840 g | -Bilateral frontal and parietal cortex | 1 Hz | 1-10 V/m | -Epicranial -Bilateral above barrel cortex -Reference on anterior midline 2-5 mm anterior from bregma | Conductive end of polyimide flex cable | X |  | -Awake rest, during spontaneous spike-wave episodes | -Tripolar electrodes for extracellular recording |

* Measurement in one monkey with closely spaced electrodes

** Estimated in a head model

**REFERENCES**

Anastassiou, C. A., R. Perin, H. Markram and C. Koch (2011). "Ephaptic coupling of cortical neurons." Nat Neurosci **14**(2): 217-223.

Berenyi, A., M. Belluscio, D. Mao and G. Buzsaki (2012). "Closed-loop control of epilepsy by transcranial electrical stimulation." Science **337**(6095): 735-737.

Bikson, M., M. Inoue, H. Akiyama, J. K. Deans, J. E. Fox, H. Miyakawa and J. G. Jefferys (2004). "Effects of uniform extracellular DC electric fields on excitability in rat hippocampal slices in vitro." J Physiol **557**(Pt 1): 175-190.

Chan, C. Y. and C. Nicholson (1986). "Modulation by applied electric fields of Purkinje and stellate cell activity in the isolated turtle cerebellum." J Physiol **371**: 89-114.

Deans, J. K., A. D. Powell and J. G. Jefferys (2007). "Sensitivity of coherent oscillations in rat hippocampus to AC electric fields." J Physiol **583**(Pt 2): 555-565.

Francis, J. T., B. J. Gluckman and S. J. Schiff (2003). "Sensitivity of neurons to weak electric fields." J Neurosci **23**(19): 7255-7261.

Frohlich, F. and D. A. McCormick (2010). "Endogenous electric fields may guide neocortical network activity." Neuron **67**(1): 129-143.

Ghai, R. S., M. Bikson and D. M. Durand (2000). "Effects of applied electric fields on low-calcium epileptiform activity in the CA1 region of rat hippocampal slices." J Neurophysiol **84**(1): 274-280.

Gluckman, B. J., E. J. Neel, T. I. Netoff, W. L. Ditto, M. L. Spano and S. J. Schiff (1996). "Electric field suppression of epileptiform activity in hippocampal slices." J Neurophysiol **76**(6): 4202-4205.

Gluckman, B. J., T. I. Netoff, E. J. Neel, W. L. Ditto, M. L. Spano and S. J. Schiff (1996). "Stochastic Resonance in a Neuronal Network from Mammalian Brain." Phys Rev Lett **77**(19): 4098-4101.

Jefferys, J. G. (1981). "Influence of electric fields on the excitability of granule cells in guinea-pig hippocampal slices." J Physiol **319**: 143-152.

Kar, K., J. Duijnhouwer and B. Krekelberg (2017). "Transcranial Alternating Current Stimulation Attenuates Neuronal Adaptation." J Neurosci **37**(9): 2325-2335.

Krause, M. R., T. P. Zanos, B. A. Csorba, P. K. Pilly, J. Choe, M. E. Phillips, A. Datta and C. C. Pack (2017). "Transcranial Direct Current Stimulation Facilitates Associative Learning and Alters Functional Connectivity in the Primate Brain." Curr Biol **27**(20): 3086-3096 e3083.

Lafon, B., A. Rahman, M. Bikson and L. C. Parra (2017). "Direct Current Stimulation Alters Neuronal Input/Output Function." Brain Stimul **10**(1): 36-45.

Maeda, K., R. Maruyama, T. Nagae, M. Inoue, T. Aonishi and H. Miyakawa (2015). "Weak sinusoidal electric fields entrain spontaneous Ca transients in the dendritic tufts of CA1 pyramidal cells in rat hippocampal slice preparations." PLoS One **10**(3): e0122263.

Ozen, S., A. Sirota, M. A. Belluscio, C. A. Anastassiou, E. Stark, C. Koch and G. Buzsaki (2010). "Transcranial electric stimulation entrains cortical neuronal populations in rats." J Neurosci **30**(34): 11476-11485.

Radman, T., Y. Su, J. H. An, L. C. Parra and M. Bikson (2007). "Spike timing amplifies the effect of electric fields on neurons: implications for endogenous field effects." J Neurosci **27**(11): 3030-3036.

Reato, D., M. Bikson and L. C. Parra (2015). "Lasting modulation of in vitro oscillatory activity with weak direct current stimulation." J Neurophysiol **113**(5): 1334-1341.

Reato, D., A. Rahman, M. Bikson and L. C. Parra (2010). "Low-intensity electrical stimulation affects network dynamics by modulating population rate and spike timing." J Neurosci **30**(45): 15067-15079.

Terzuolo, C. A. and T. H. Bullock (1956). "Measurement of Imposed Voltage Gradient Adequate to Modulate Neuronal Firing." Proc Natl Acad Sci U S A **42**(9): 687-694.

Voroslakos, M., Y. Takeuchi, K. Brinyiczki, T. Zombori, A. Oliva, A. Fernandez-Ruiz, G. Kozak, Z. T. Kincses, B. Ivanyi, G. Buzsaki and A. Berenyi (2018). "Direct effects of transcranial electric stimulation on brain circuits in rats and humans." Nat Commun **9**(1): 483.

Xu, W., B. S. Wolff and J. Y. Wu (2014). "Low-intensity electric fields induce two distinct response components in neocortical neuronal populations." J Neurophysiol **112**(10): 2446-2456.
